# Late Recurrence in Ovarian Dysgerminoma Presenting as a Primary Retroperitoneal Tumor: A Case Report and Review of the Literature

**DOI:** 10.1155/2020/4737606

**Published:** 2020-02-13

**Authors:** Yuichiro Sato, Tohru Hayashi, Hidetaka Yamamoto, Ichiro Niina, Naoya Kuroki, Takeshi Iwamura, Junji Onishi

**Affiliations:** ^1^Department of Diagnostic Pathology, Miyazaki University Hospital, University of Miyazaki, 5200 Kihara, Kiyotake, Miyazaki 889-1692, Japan; ^2^Department of Diagnostic Pathology, Junwakai Memorial Hospital, Komatsu, Miyazaki 880-2112, Japan; ^3^Department of Clinical Laboratory, Breastopia Miyazaki Hospital, Maruyama, Miyazaki 880-0052, Japan; ^4^Department of Anatomic Pathology, Pathological Sciences, Graduate School of Medical Sciences, Kyusyu University, Umade, Fukuoka 812-8582, Japan; ^5^Department of Surgery, Junwakai Memorial Hospital, Komatsu, Miyazaki 880-2112, Japan; ^6^Department of Obstetrics and Gynecology, Faculty of Medicine, University of Miyazaki, Miyazaki, 5200 Kihara, Kiyotake, Miyazaki 889-1692, Japan

## Abstract

Ovarian dysgerminoma is a rare type of germ cell tumor. The majority of patient relapses occur within 2 years of diagnosis. Here, we report the case of a 74-year-old woman with a history of ovarian dysgerminoma 39 years earlier. The patient visited the hospital presenting with heartburn. An abdominal computed tomography (CT) revealed a right retroperitoneal mass, and a primary retroperitoneal tumor was suspected. She underwent surgical resection of the retroperitoneal tumor. Histological examination confirmed a metastatic dysgerminoma to the retroperitoneum. Postoperative CT showed paraaortic and cervical lymph node metastases. The patient was treated with bleomycin, etoposide, and cisplatin chemotherapy. This case demonstrates the difficulties that may be encountered in the differential diagnosis of a retroperitoneal mass and underlines the necessity for understanding a patient's clinical history.

## 1. Introduction

Dysgerminoma of the ovary is a rare type of primitive germ cell tumor accounting for 1-2% of all ovarian neoplasms. Dysgerminoma is composed entirely of germ cells that show morphologic and histochemical similarity to primordial germ cells. Most cases occur in the second and third decades; nearly half of the patients are under 20 years of age. Eighty-five percent of patients with dysgerminoma present with unilateral disease, and the majority of patients have stage Ia disease at the time of diagnosis [1]. Dysgerminoma is a malignant neoplasm capable of metastatic and local spread. Relapses usually occur within 2 years of diagnosis and the recurrence rate is approximately 10-20%, and it has been reported that more than 75% occur in the first year [2–4]. To date, only a handful of cases of late recurrence of ovarian dysgerminoma have been reported in the English language literature [5–11]. Here, we describe a case of a female patient with a late recurrence of ovarian dysgerminoma, after 39 years, presenting a primary retroperitoneal tumor.

## 2. Case Presentation

**Figure 1:**
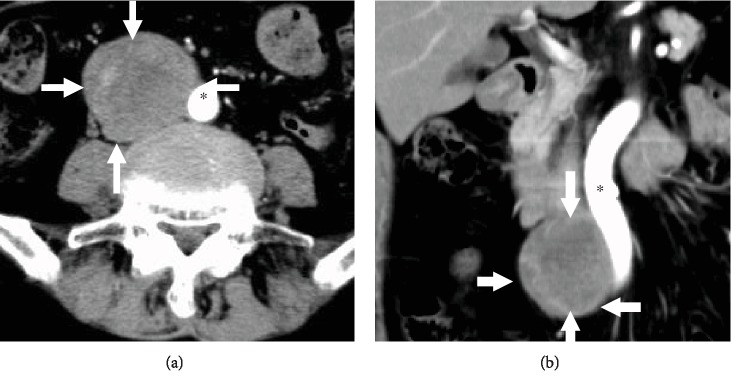
Computed tomography (CT) images at the recurrence revealed a large tumor (arrows) in the retroperitoneum, which was crossed to the aorta (^∗^). (a) Horizontal section and (b) coronal section.

A 74-year-old female (gravida 3, para 2) was initially treated 39 years previously (at 35 years of age) for ovarian dysgerminoma. She underwent a bilateral oophorectomy hysterectomy and was treated with radiation therapy. The patient presented at the hospital with heartburn. An abdominal computed tomography (CT) revealed a right retroperitoneal mass (5 × 4 cm) (Figure 1). This mass was located close to the aorta and duodenum. There were several swollen lymph nodes in the paraaortic lesion, but no involvement of the other organ. A gastrointestinal stromal tumor (GIST), ganglioneuroma, and other primary retroperitoneal tumors were suspected by radiological examination. Surgical resection of the retroperitoneal tumor was then performed.

**Figure 2:**
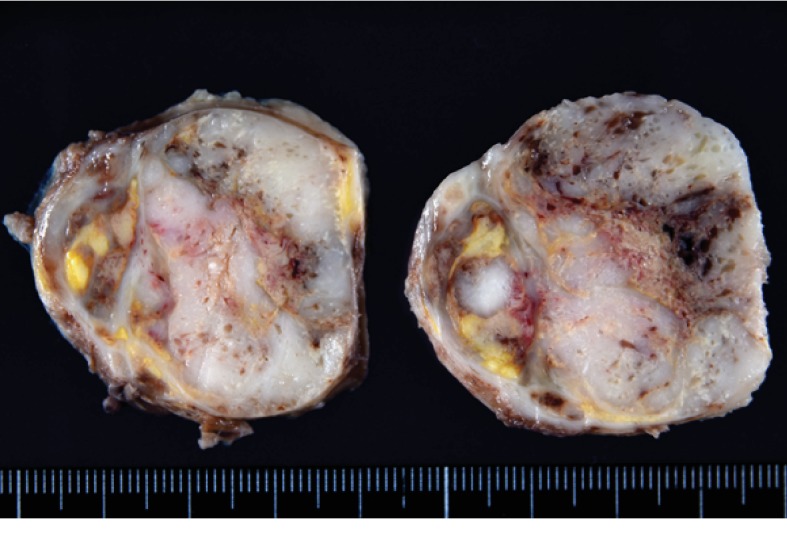
Macroscopic findings for the retroperitoneal mass. The cut surface showed a relatively circumscribed tumor. Hemorrhage and necrosis were present.

**Figure 3:**
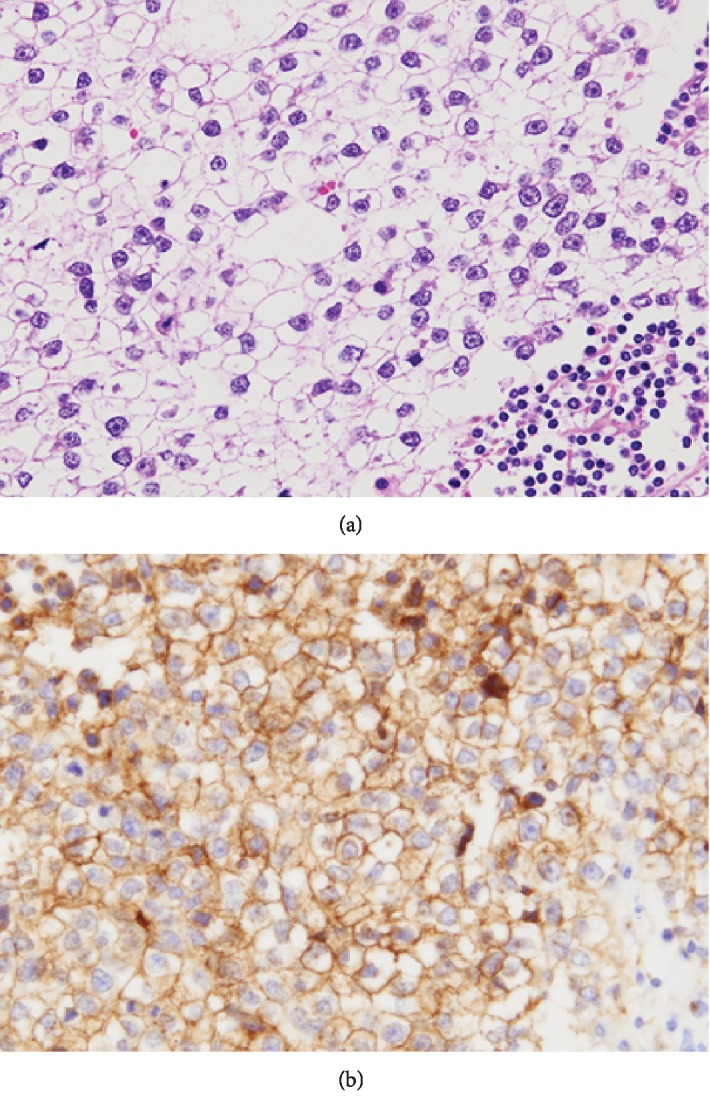
Microscopical findings for the retroperitoneal tumor. (a) Polygonal tumor cells proliferated in sheets or nests with lymphocytes infiltration. (b) Immunohistochemical study for D2-40. Strong membranous D2-40 expression was present.

The resected tumor was a relatively well-circumscribed ovoid mass, measuring 6 × 5 × 5 cm. The cut surface was gray to white with hemorrhage and necrosis (Figure 2). Histologically, polygonal tumor cells proliferated diffusely or in sheets (Figure 3(a)). These tumor cells had a clear or eosinophilic cytoplasm and large round nuclei with prominent nucleoli. Lymphocytic infiltration was also present. Histological differential diagnoses were adrenal carcinoma, GIST, perivascular epithelioid cell tumor (PEComa), or dysgerminoma, due to the pathologists having no information of previous dysgerminoma at the initial pathological diagnosis. Immunohistochemically, these tumor cells were positive for D2-40 (Figure 3(b)), OCT3/4, and SALL4, but negative for c-kit, AE1/AE3, CK18, DOG1, S-100, HMB-45, and Melan A. The pathological diagnosis was dysgerminoma. After this diagnosis, the pathologists obtained the information of previous dysgerminoma; thus, the final diagnosis was confirmed as the recurrence of ovarian dysgerminoma. Postoperative CT revealed paraaortic and cervical lymph nodes metastases. The patient was treated with bleomycin, etoposide, and cisplatin chemotherapy.

## 3. Discussion

Dysgerminoma of the ovary is a rare germ cell tumor accounting for 1-2% of all ovarian neoplasms and only 3-5% of ovarian malignancies. These tumors tend to occur at a young age with a median age of 20 years at diagnosis. It is usually curable with appropriate surgery, radiation, or chemotherapy. Around 10-20% of these patients have a recurrence of tumors, with the majority recurring within the first 2 years [1–3].

When young women have an early stage ovarian tumor, many investigators have recommended they are treated with unilateral salpingo-oophorectomy [12]. Before the era of combination chemotherapy, most patients were treated with radiotherapy, including radiotherapy for the pelvis and paraaortic nodes. At the initial surgery, the patient visited a previous hospital for artificial abortion and abdominal mass resection. She underwent bilateral salpingo-oophorectomy and hysterectomy. She also received abdominal radiation therapy, without any adjuvant chemotherapy. Her disease recurred after 39 years with a retroperitoneal tumor. Ovarian dysgerminoma is extremely chemotherapy sensitive [12, 13]. The patient received chemotherapy for the recurrence of the disease.

A report on the late recurrence (more than 2 years after primary treatment) revealed this to be a rare entity (Table 1). Only 9 cases of late recurrence have been reported. Six of the 9 cases were more than 2 years but less than 10 years post primary tumor, and 3 cases were more than 10 years. The most common recurrence sites were the pelvis and abdomen, including retroperitoneum and paraaortic lymph nodes. Seven patients were initially treated with surgery alone, and 5 were treated by chemotherapy at the recurrence. Four patients (44%) with late recurrence died. The prognosis of a patient with late recurrence was worse than that of a patient with recurrence within 2 years [4, 5], probably due to widespread disease and a low efficacy rate of chemotherapy. This case emphasized the need for continued long-term follow-up of all patients with dysgerminoma.

The differential diagnosis of soft tissue masses in the retroperitoneum includes metastatic carcinoma, lymphoma, and soft tissue sarcomas. GIST was also clinically suspected in this case, due to the location of the tumor being near the duodenum. We suspected a poorly differentiated carcinoma, GIST, PEComa, or dysgerminoma by histological findings, because there was no information on the patient history at initial diagnosis. Immunohistochemical analysis confirmed dysgerminoma. Subsequently, we suspected a primary retroperitoneal dysgerminoma or metastatic dysgerminoma. There were few reports of primary retroperitoneal dysgerminoma [14, 15]. In this case, the pathologists had access to the patient's clinical history of ovarian dysgerminoma after initial diagnosis and then the final diagnosis of metastasis from ovarian dysgerminoma was confirmed.

## 4. Conclusions

We have described the latest recurrent case of an ovarian dysgerminoma. This case demonstrated the difficulties that may be encountered in the differential diagnosis of a primary retroperitoneal tumor. The clinical history of a patient and the collaboration between clinicians and pathologists are essential for an accurate diagnosis.

## Figures and Tables

**Table 1:** Reported and present cases of late recurrence (more than 2 years) in ovarian dysgerminomas.

Case number	Patient age at recurrence (years old)	Time of late recurrence	Initial treatment	Site recurrence	Treatment at late recurrence	Outcome
1 [5]	26	6 years	Surgery	Neck and abdomen	Surgery	Died
2 [6]	58	33 years	Surgery	Paraaortic, supraclavicular LN	Surgery, chemo	Alive
3 [7]	29	2.5 years	Surgery, RT	Abdomen	None	Died
4 [7]	31	5.7 years	Surgery	Abdomen	None	Died
5 [8]	Not available	5 years	Surgery	Paraaortic LN	Surgery	Alive
6 [9]	12	5 years	Surgery	Peritoneum	Chemo, RT	Alive
7 [9]	19	6 years	Surgery, chemo	Pelvis and retroperitoneum	Surgery, chemo	Alive
8 [10]	32	20 years	Surgery	Pelvis and abdomen	Chemo	Died
9 [11]	37	12 years	Surgery	Pelvis	Chemo	Alive
10 (present case)	74	39 years	Surgery, RT	Retroperitoneum	Surgery, chemo	Alive

LN: lymph node; RT: radiation therapy; chemo: chemotherapy.
